# Evaluating Infection Prevention Strategies in Out-Patient Dialysis Units Using Agent-Based Modeling

**DOI:** 10.1371/journal.pone.0153820

**Published:** 2016-05-19

**Authors:** Joanna R. Wares, Barry Lawson, Douglas Shemin, Erika M. C. D’Agata

**Affiliations:** 1 Department of Mathematics and Computer Science, University of Richmond, Richmond, Virginia, United States of America; 2 Division of Nephrology, Rhode Island Hospital, Brown University, Providence, Rhode Island, United States of America; 3 Division of Infectious Diseases, Rhode Island Hospital, Brown University, Providence, Rhode Island, United States of America; Southwest University, CHINA

## Abstract

Patients receiving chronic hemodialysis (CHD) are among the most vulnerable to infections caused by multidrug-resistant organisms (MDRO), which are associated with high rates of morbidity and mortality. Current guidelines to reduce transmission of MDRO in the out-patient dialysis unit are targeted at patients considered to be high-risk for transmitting these organisms: those with infected skin wounds not contained by a dressing, or those with fecal incontinence or uncontrolled diarrhea. Here, we hypothesize that targeting patients receiving antimicrobial treatment would more effectively reduce transmission and acquisition of MDRO. We also hypothesize that environmental contamination plays a role in the dissemination of MDRO in the dialysis unit. To address our hypotheses, we built an agent-based model to simulate different treatment strategies in a dialysis unit. Our results suggest that reducing antimicrobial treatment, either by reducing the number of patients receiving treatment or by reducing the duration of the treatment, markedly reduces overall colonization rates and also the levels of environmental contamination in the dialysis unit. Our results also suggest that improving the environmental decontamination efficacy between patient dialysis treatments is an effective method for reducing colonization and contamination rates. These findings have important implications for the development and implementation of future infection prevention strategies.

## Introduction

Rates of multidrug-resistant organisms (MDRO) are among the highest in the population of chronic hemodialysis (CHD) [1–3]. Infections caused by MDRO are associated with considerable morbidity and mortality, and limited therapeutic options [4–6]. It is therefore imperative to curtail the ongoing spread and *de novo* acquisition of MDRO in the CHD population. In out-patient dialysis units, current guidelines recommend that only those patients at high-risk of disseminating MDRO be placed on additional infection control precautions. These precautions include the use of a separate gown by the healthcare worker (HCW) with removal of gown when finished caring for the patient, as well as dialyzing the patient at a station with the fewest adjacent stations [7]. These high-risk patients are defined as those with infected skin wounds not contained by a dressing, or those with fecal incontinence or uncontrolled diarrhea. These recommendations are extrapolated from hospital-based studies and their efficacy in preventing MDRO spread within the unique out-patient dialysis setting has not been quantified.

Another subgroup of CHD patients that may be at higher risk of MDRO dissemination are colonized patients who are receiving antimicrobials. It is well-established that antimicrobial exposure leads to increased MDRO bacterial loads in the gastrointestinal tract and nares [8–10]. Higher bacterial loads result in greater skin contamination and thus greater likelihood of transmission to healthcare workers and other patients [11]. Additionally, increased MDRO loads have also been directly associated with greater environmental contamination.[8] Furthermore since antimicrobial treatment reduces the endogenous flora that naturally out-competes MDRO, patients receiving antimicrobial treatment are the group most likely to become colonized with MDRO [12]. Thus, CHD patients who are receiving antimicrobials may be another subgroup of patients at high-risk of MDRO spread.

Specific precautions targeting CHD patients who are receiving antimicrobials may therefore be warranted. To test this hypothesis, we quantified and compared the transmission dynamics of MDRO in out-patient dialysis units between those high-risk patients targeted by current recommendations and those patients colonized with MDRO who are receiving antimicrobials. Given the numerous interrelated and dynamic factors contributing to MDRO transmission and the small number of patients in dialysis units, agent-based modeling was used, since classic epidemiological studies cannot fully address the complexities of transmission. Environmental and healthcare worker contamination, compliance with hand hygiene, and extent of antimicrobial exposure were also included in the model, and their role in MDRO transmission quantified.

## Materials and Methods

### Simulation Model

An agent-based simulation model was developed to study the transmission dynamics of MDRO in an out-patient dialysis unit. The goal is to recognize transmission patterns that emerge at the dialysis-unit level as a result of the definition of individual-level behaviors. Agent-based models consist of a collection of autonomous, heterogeneous agents (individuals), an environment in which the agents reside, and a collection of rules that govern how the agents interact individually with one another and with the environment. Each agent has a set of characteristics (attributes) whose values vary across time and are unique to that agent, as well as a set of behaviors (actions) that the agent performs. The environment may also have attributes. In the model presented here, the environment is the out-patient dialysis unit, specifically a collection of chairs where patients receive dialysis. The model uses two types of agents, representing the patients receiving dialysis and the healthcare workers (HCWs) caring for those patients.

Our model dialysis unit provides care to 120 patients. Each patient receives three-hour dialysis sessions three times per week, on either a Monday/Wednesday/Friday schedule or a Tuesday/Thursday/Saturday schedule, and remains on the same schedule for a two-year period. Each day consists of four shifts, each lasting four hours. Patients receive treatment during the same shift on each of their treatment days. There are five HCWs per shift and each HCW works two consecutive shifts, treating three patients per shift, and is presumed to be without MDRO contamination at the start of their first of the two shifts. Each patient interacts with their HCW at the beginning and at the end of a dialysis session, and may be revisited by the HCW during the session (on average two additional times per session). Before each interaction with a patient, a proportion of HCWs successfully comply with hand hygiene guidelines. Patients start antimicrobial treatments once per year on average, and are admitted to the hospital twice per year on average, where they may acquire *de novo* MDRO colonization and/or receive antimicrobials [3,13,14]. The following MDRO transmission routes within the dialysis unit are considered:

- contamination of a HCW by a colonized patient;
- contamination of a chair by a colonized patient;
- colonization of a patient on antimicrobials via contact with a contaminated HCW; and
- colonization of a patient on antimicrobials via contact with a contaminated chair.

Additionally, a patient returning to the dialysis unit from a hospital visit may have *de novo* MDRO colonization as a result of the hospitalization.

Attributes of the environment and the agents in the model are defined to be consistent with these transmission routes. Chairs and HCWs each have one defined attribute: MDRO contamination status. For patients, we define four attributes: (a) MDRO colonization status; (b) antimicrobial treatment status; (c) super-spreader (SS) status; and (d) hospital admission status. In this context, SS are defined as the group of colonized patients at higher risk of MDRO transmission, with two corresponding categorizations: 1) an “SS1” patient is defined as a colonized patient meeting the CDC guidelines for “high-risk” of MDRO transmission and 2) an “SS2” patient is defined as a colonized patient receiving antimicrobial treatment [7]. Note that SS2 patients that are also categorized as being SS1 (i.e., are colonized with MDRO, receiving antimicrobial treatment, *and* meet the CDC guidelines for “high-risk”) transmit bacteria at a higher rate consistent with being SS1.

Agent behaviors are also modeled to be consistent with the transmission routes and attributes discussed above. Our agent-based simulation model uses an *event-oriented view* to model time. Therefore, we define a collection of event types corresponding to agent behaviors in the model, where the occurrence of any event (of one of those types) may change the state of our simulated out-patient dialysis unit. As an example, one event type corresponds to a HCW visiting a patient mid-session, potentially resulting in a change in the number of colonized patients (if the HCW is contaminated and transmits MDRO to the patient). We define 15 different types of events in the model, given in Table 1.

**Table 1:** Event Types for the Dialysis Unit Model.

1) HCW begins shift	8) patient begins antimicrobial treatment
2) HCW ends shift	9) patient ends antimicrobial treatment
3) patient begins dialysis session	10) patient becomes colonized
4) patient ends dialysis session	11) patient becomes no longer colonized
5) HCW visits patient (mid-session)	12) patient becomes SS1
6) HCW cleans the chair (post-session)	13) patient ends being SS1
7) HCW is hand-hygiene compliant (pre-visit)	14) patient enters hospital
	15) patient leaves hospital

During execution, the simulation model maintains a calendar (list) of events to occur chronologically in simulated time. After an initial list of events is generated, the simulation advances according to the following sequence of steps: (a) the next event to occur in simulated time is fetched from the event calendar; (b) the simulation clock is advanced to the time of that event; (c) an algorithm handling that particular type of event is executed; and (d) the calendar is updated appropriately (e.g., to include a future event corresponding to the type of event just handled). This sequence of steps is repeated, updating the simulation clock and event calendar each time, until the clock reaches the maximum time to simulate. In this way, time is modeled asynchronously as the simulation clock advances non-uniformly to discrete points in time, rather than synchronously using a fixed time step. Because events of interest in the dialysis unit occur asynchronously in time in practice, modeling time asynchronously rather than synchronously facilitates a more realistic representation of the times at which events occur, and is more computationally efficient, which is of particular importance for large-scale state-space exploration of parameter values. Our model was implemented in MATLAB (The MathWorks, Inc.; Natick, MA, USA), using object-oriented programming to implement the agents (patients and HCWs), environment (chairs), and the event-oriented simulation engine.

### Treatment Schedule and Model Parameters

At the beginning of every shift, patients are randomly assigned to dialysis stations (chairs) in the unit. HCWs are then assigned to a group of three patients. Each patient is treated, in no particular order, by a HCW at the beginning and end of the patient’s dialysis session. On average, each patient is seen two additional times during the session. Before each patient visit, HCWs successfully follow hand hygiene guidelines 50% of the time.

Patients start antimicrobial treatments once a year on average with treatment duration lasting three weeks [13]. Hospital admissions occur twice a year per patient on average with a mean length of stay of 11 days [14]. Patients have a 20% probability of leaving the hospital with *de novo* MDRO acquisition [9]. During the hospitalization, on average 25% of patients begin an antimicrobial course that continues for two more weeks in the dialysis unit [13].

Values for parameters governing transmission between patients and HCWs in the dialysis unit were obtained from the literature and from fitting the model to the values for non-transmission parameters given in Table 2. Baseline values for these parameters are assigned such that during each patient visit, HCWs become contaminated with a probability of 40% when coming into contact with an SS2, and with a probability of 20% if the patient is colonized with MDRO but not receiving antimicrobial treatment [11]. Contaminated HCWs (who remain contaminated as a result of non-compliance with hand hygiene measures) transmit MDRO to uncolonized patients on antimicrobials with a probability of 6% [15]. On average, patients that become colonized with MDRO remain colonized for 12 weeks [16].

**Table 2:** Non-Transmission Model Parameters with Baseline Estimates.

Parameter	Value	Reference
Number of patients in out-patient dialysis unit	120	N/A
Initial percent of patients colonized with MDRO	15%	16
Initial percentage of colonized patients that are SS1	1%	13
Initial percentage of patients receiving antimicrobial treatment	6%	13
Initial percentage of chairs contaminated with MDRO	10%	2
Compliance with hand hygiene (%)	50%	16
Frequency of antimicrobial courses	1 per year	13
Duration of antimicrobial course	3 weeks	13
Frequency of hospital admission per patient	2 per year	14
Mean length of hospital stay	11 days	14
Probability of acquiring MDRO during hospital admission (%)	20%	9
Percent of patients continuing on antimicrobials in dialysis unit after hospital discharge / duration of antimicrobials in dialysis unit	25% 2 weeks	13

Among those patients that become colonized, on average 10% will become SS1 [13] while colonized and remain “high risk” for 2 weeks [13]. CDC-defined high-risk patients (SS1) were assumed to be 50% more likely to transmit MDRO than patients that are colonized and receiving antimicrobial treatment (SS2): 60% probability for SS1 and 40% probability for SS2. Sensitivity analyses were performed on all transmission parameters. Non-transmission parameters are presented in Table 2.

Patients receiving antimicrobial treatment can also become colonized with MDRO by coming into contact with MDRO-contaminated chairs. An extensive literature search provided no established values for the transmission probability parameters from a contaminated chair to a patient, or from a colonized patient to a chair. Accordingly, values for the parameters corresponding to those probabilities were determined empirically using our simulation model via a two-dimensional search of parameter values so that outcome measures matched data known from literature.

To fit the parameter values, we executed the simulation model using a range of 0% to 100% (in 5% increments) for each of the patient-to-chair and chair-to-patient transmission probabilities, varying the values of the two transmission probabilities together. We identified those parameter values that resulted in outcome measures from the simulation that were within 1% of the expected values given above from the literature, resulting in four possible choices of parameter value pairs (chair to patient: patient to chair): 40%:60%, 45%:55%, 50%:50%, 55%:45%. The simplest parameter value pairs were chosen: (a) 50% transmission probability from a contaminated chair to a patient and (b) 50% transmission probability from a colonized patient to a chair. All results presented below used values of 50% and 50% for these two parameters in the corresponding experiments. We also executed the same set of experiments using parameter values of 40% (chair-to-patient transmission) and 60% (patient-to-chair). All results were qualitatively similar to those produced using 50% and 50%.

### Outcome Measures

All results presented below correspond to a two-year long simulation. Starting from the baseline values, we systematically varied the value of only one parameter at a time, generating 500 replications (two-year simulation runs) for each set of parameter values. After the occurrence of any event in a replication, the number of (a) patients colonized with MDRO, (b) SS1, (c) SS2, (d) patients receiving antimicrobials, and (e) contaminated chairs were recorded, along with the corresponding time of the event. The outcome measures were computed as the time-averaged percentage, across all 500 replications, of each of these five groups over only the second year of simulated time (removing transient effects of initial conditions).

## Results

### Baseline Experiment

The first goal of this study was to benchmark the simulation model to match data values documented in the literature. Simulation experiments produced the outcome measures given in Table 3. Appropriately, the outcome measures were within 1% of the data values cited in the literature. This benchmarking provides confidence in additional experimentation presented below involving systematic exploration of model parameters and the corresponding effects.

**Table 3:** Baseline Outcome Measures.

	Average Percent	95% Confidence Interval
**Patients Colonized**	14.5%	[14.2%, 14.7%]
**SS1**	0.17%	[0.16%, 0.18%]
**SS2**	1.94%	[1.91%, 1.98%]
**Contaminated Chairs**	9.0%	[8.9%, 9.2%]
**Patients Receiving Antimicrobial Treatment**	7.0%	[7.0%, 7.1%]

### Simulation 1A: Varying transmission from SS1 to HCW

**Fig 1:**
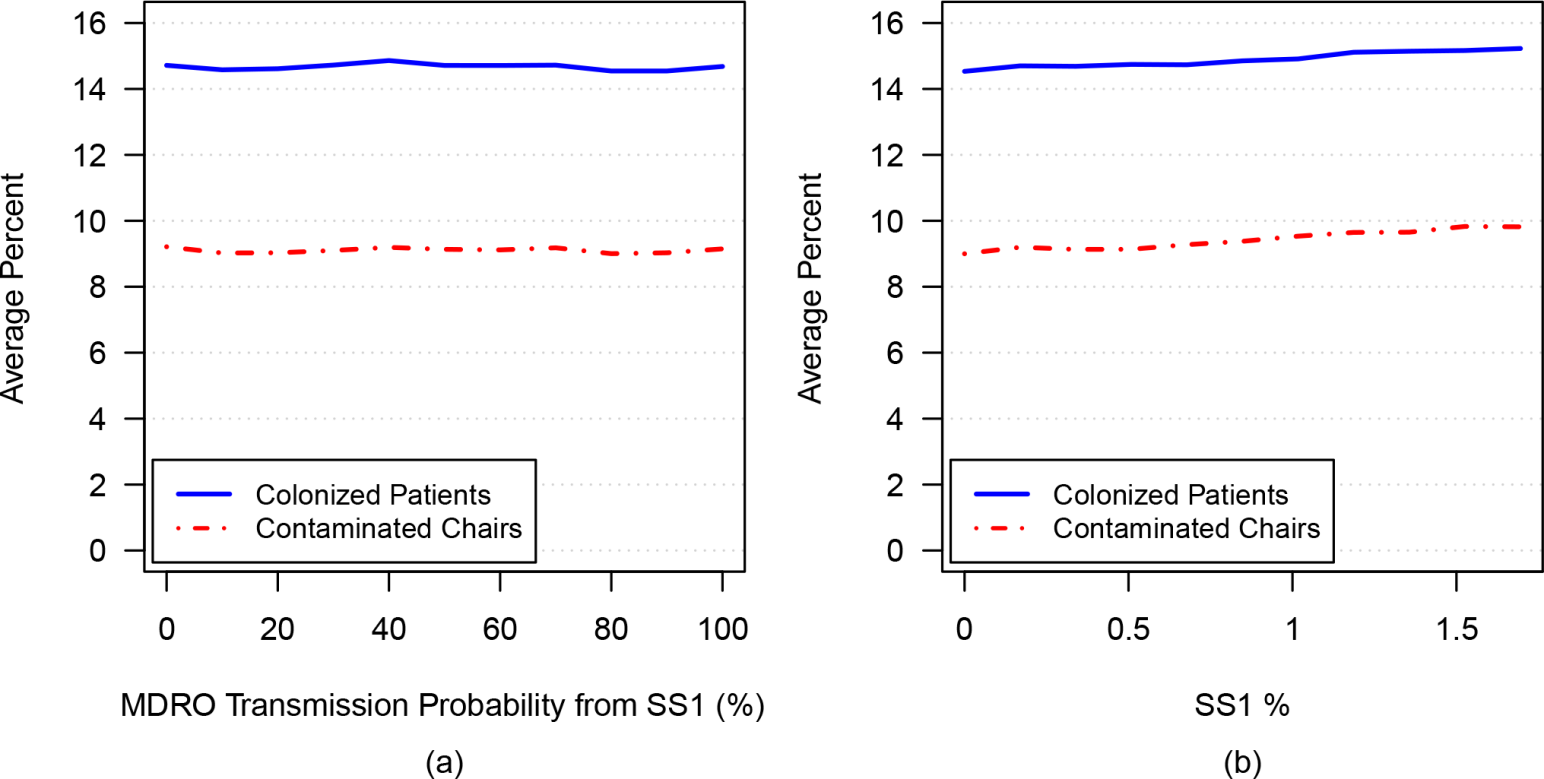
The average percentages of MDRO-colonized patients (solid blue curve) and MDRO-contaminated chairs (dashed red curve) are plotted versus: a) increasing transmission probability from SS1 to HCW; b) increasing percentage of SS1.

To determine the effect of increased transmission from SS1 to HCW populations, the transmission probability resulting from contact between an SS1 patient and an uncontaminated HCW was varied from 0% to 100%. As shown in Fig 1A, no substantive change in the total percentage of MDRO-colonized patients (solid blue line) or MDRO-contaminated chairs (dashed red line) occurred as the transmission probability increased. The percentage of MDRO-colonized patients varied negligibly with increased transmission probability, being 14.7% on average with a 95% confidence interval of [14.5%, 14.9%] at both the 0% and 100% probability extremes.

### Simulation 1B: Varying the likelihood of colonized patients becoming SS1

To further investigate the effect of the SS1 patient population on overall colonization and contamination rates in the unit, the probability of MDRO colonization among the subgroup of patients at risk of becoming SS1 (uncovered wound, fecal incontinence or diarrhea), after *de novo* colonization, was varied from 0% to 100%. This range of probabilities resulted in the population of SS1 patients increasing from 0% to 1.7% (of all patients) on average at any time, corresponding to 0 to 2 SS1 patients at any time in the dialysis unit.

Fig 1B depicts the percentage of colonized patients and percentage of contaminated chairs versus the percentage of SS1 patients (of the total patient population). In this figure, the percentage of SS1 patients increased from 0% to 1.7% along the horizontal axis, a result of increasing the probability of colonization occurring among the subgroup of patients at risk of becoming SS1 from 0% to 100%. As shown in the figure, as the population of SS1 increases, a small increase in colonized patients, from 14.5% to 15.2% on average, and in contaminated chairs, from 9% to 9.8% on average, occurs.

### Simulation 2A: Varying transmission from SS2 to HCW

**Fig 2:**
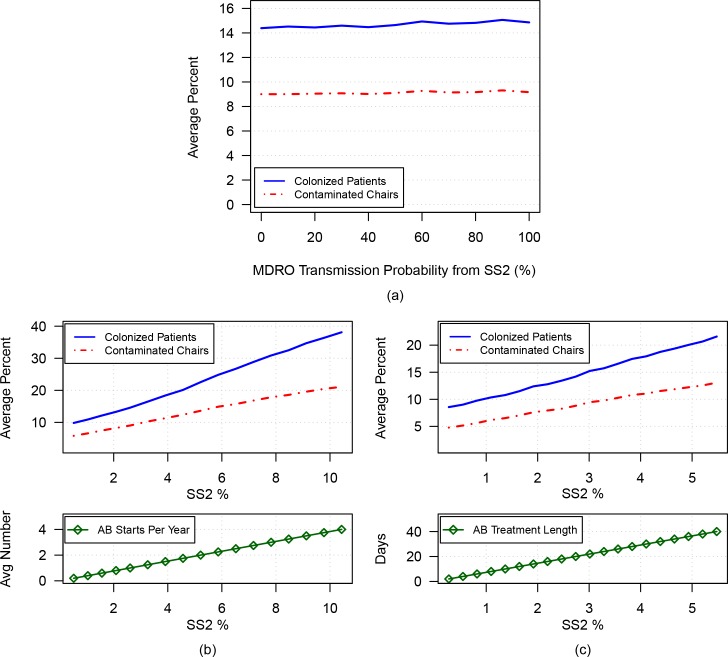
The average percentages of MDRO-colonized patients (solid blue curve) and MDRO-contaminated chairs (dashed red curve) are plotted versus: a) increasing transmission probability from SS2 to HCW; b) increasing percentage of SS2 (a result of increasing the average number of antimicrobial treatments per year from 0 to 4); c) increasing percentage of SS2 (a result of increasing the duration of antimicrobial treatment from 2 to 40 days).

Next, the transmission probability resulting from contact between an SS2 patient and an uncontaminated HCW was varied from 0% to 100%. As shown in Fig 2A, as the transmission probability from SS2 to HCW increases, there is a small increase in the percentage of MDRO-colonized patients (solid blue line) and percentage of MDRO-contaminated chairs (dashed red line). At the 0% and 100% transmission probability extremes, the average percentage of MDRO-colonized patients is 14.4% and 14.9% respectively, with corresponding 95% confidence intervals of [14.2%, 14.6%] and [14.6%, 15.1%], respectively.

### Simulations 2B and 2C: Varying the likelihood of colonized patient becoming SS2 by varying the frequency and duration of antimicrobials

The results in Fig 2A indicate that the transmission rate from SS2 to HCW has a minimal effect when the number of patients in the SS2 population is small. However, when the parameters governing antimicrobial treatment are varied, the size of the SS2 population changes significantly. In addition, as the SS2 population size increases with increased usage and duration of antimicrobials, colonization and contamination rates increase dramatically. Fig 2B considers frequency of antimicrobial use. The upper plot of Fig 2B depicts the average percentage of colonized patients and contaminated chairs versus the percentage of SS2 patients (of the total patient population). In this figure, the percentage of SS2 patients increases from 0.7% to 10.4% along the horizontal axis, driven by an increase in antimicrobial treatments from once every year to four times per year on average (depicted in the lower plot). Correspondingly, the percentage of MDRO-colonized patients increases from 9.8% to 38.1%, and the percentage of MDRO-contaminated chairs increases from 5.8% to 21.1%. Although not shown in the figure, the percentage of SS1 increases only from 0.12% to 0.43% of all patients.

Because longer antimicrobial treatments correspond to more patients receiving treatment at any one time (therefore affecting the size of the SS2 population), we also investigated the duration of antimicrobial use. The upper plot of Fig 2C depicts the average percentage of colonized patients and contaminated chairs versus the percentage of SS2 patients (of the total patient population). In this figure, the percentage of patients that are SS2 increases from 0.1% to 5.5% along the horizontal axis, driven by an increase in antimicrobial duration from 2 to 40 days (depicted on the vertical in the lower plot). For any patient with antimicrobial treatment initiated during a hospital visit, the length of remaining treatment while in the dialysis unit was modelled as 2/3 of the duration of treatment initiated in the dialysis unit [13]. Correspondingly, the percentage of MDRO-colonized patients increases from 8.6% to 21.6%, and the percentage of MDRO-contaminated chairs increases from 4.8% to 13.1% (see upper plot). Similarly to when varying the frequency of use, the SS1 population increases minimally (from 0.09% to 0.23% of all patients).

### Simulation 3A: Varying environmental contamination

**Fig 3:**
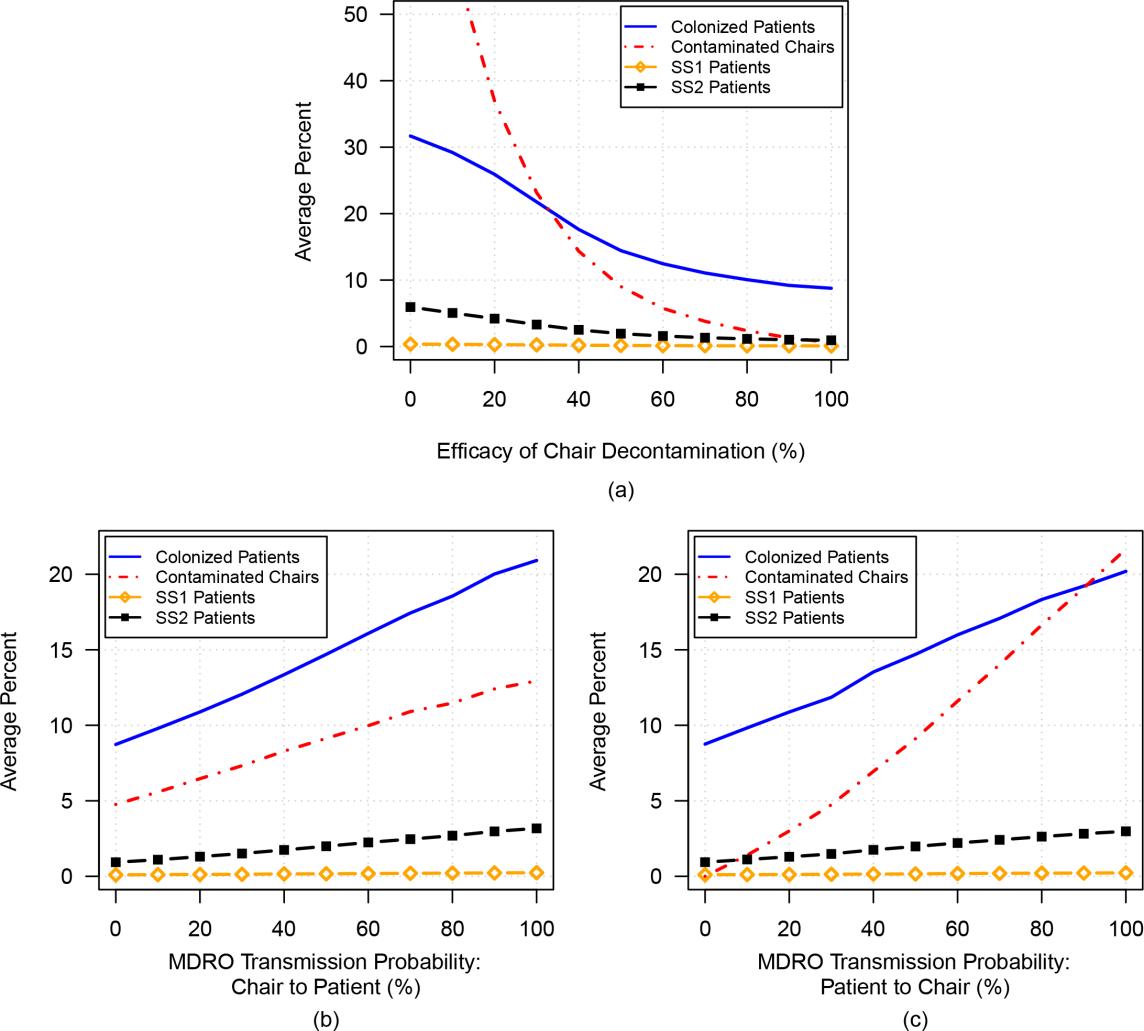
The average percentages of MDRO-colonized patients (solid blue curve), MDRO-contaminated chairs (dashed red curve), SS1 patients (gold curve with open diamonds), and SS2 patients (black curve with filled squares) are plotted versus: a) increasing efficacy of chair-decontamination efficacy; b) increasing transmission probability from chairs to patients; and c) increasing transmission probability from patients to chairs.

To determine the effect of environmental decontamination on transmission in the unit, we varied the parameter corresponding to efficacy of chair decontamination from 0% to 100%. As shown in Fig 3A, the overall percentage of MDRO-colonized patients (solid blue curve) decreases from 31.7% to 8.8% as chair-decontamination efficacy increases, with minimal effect on the SS1 population.

Additionally, sensitivity analysis was performed on the parameters governing transmission of MDRO between patients and chairs (see Fig 3B and 3C). As the probability that patients are colonized by sitting in a contaminated chair increases from 0% to 100%, the average percentage of MDRO-colonized patients increases from 8.7% to 20.9% (Fig 3B). Similarly, as the probability that a chair is contaminated by a colonized patient increases from 0% to 100%, the average percentage of MDRO-colonized patients increases from 8.8% to 20.2% (Fig 3C). As expected, an increase in the percentage of MDRO-contaminated chairs is also observed in both cases.

### Simulation 4A-C: Varying the rate of MDRO acquisition during a hospital admission

**Fig 4:**
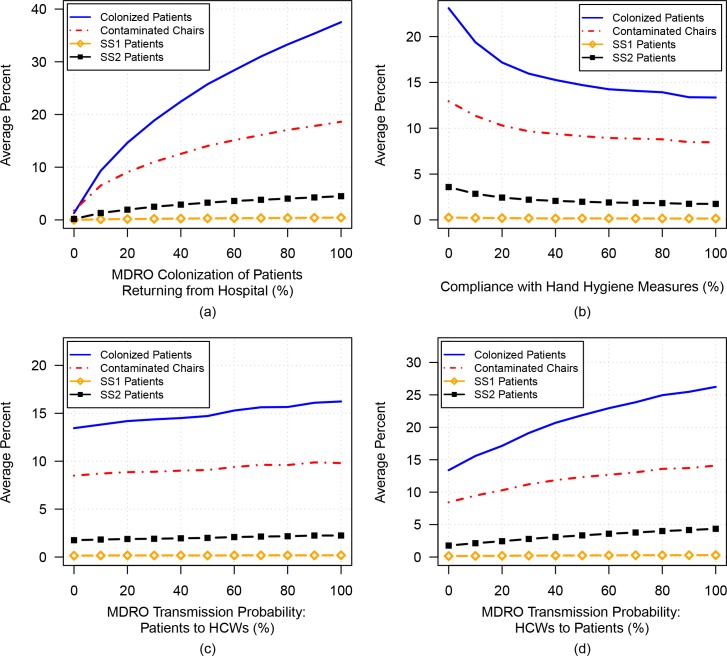
The average percentages of MDRO-colonized patients (solid blue curve), MDRO-contaminated chairs (dashed red curve), SS1 patients (gold curve with open diamonds), and SS2 patients (black curve with filled squares) are plotted versus: a) increasing probability of hospital-acquired colonization; b) increasing compliance with hand-hygiene protocol; c) increasing transmission probability from patients to HCWs; and d) increasing transmission probability from HCWs to patients.

To investigate the effects of hospital admission where *de novo* MDRO acquisition can occur, the probability of becoming MDRO-colonized during a hospitalization was varied. As shown in Fig 4A, a large increase in the percentage of MDRO-colonized patients in the dialysis unit, as well as a noticeable increase in the SS2 population, results from increasing the likelihood of hospital-acquired colonization. Again, note that the size of the SS1 population remains low throughout.

### Simulation 4B: Varying hand-hygiene compliance

Fig 4B shows the effect of increasing compliance with hand-hygiene protocols and the corresponding decrease in the percentages of MDRO-colonized patients, SS2 patients, and MDRO-contaminated chairs. Without effective hand-hygiene measures (0% efficacy), the percentage of MDRO-colonized patients increases from 14.5% at baseline to 23.1%.

### Simulation 4C and D: Varying patient-to-HCW and HCW-to-patient transmission

As shown in Fig 4C, there is a noticeable increase in the percentages of colonized patients and of contaminated chairs as patient-to-HCW transmission probability increases. A small increase in the percentage of SS2 patients is also evident, while the SS1 population remains very small. Similar results are obtained when increasing the transmission probability from HCW to an uncolonized patient (as shown in Fig 4D).

## Discussion

Current national recommendations for limiting the spread of MDRO among CHD patients target the subgroup of patients at highest-risk of MDRO dissemination: those MDRO colonized patients with open wounds, fecal incontinence or uncontrolled diarrhea, which we term superspreaders1 (SS1). In this study, we hypothesized that there is an additional subgroup of high-risk patients: those patients that are MDRO colonized and are receiving antimicrobials, which we term superspreaders2 (SS2). Numerous studies have shown that exposure to antimicrobials results in an increased MDRO bacterial load in the nares and gastrointestinal tract, and leads to substantially greater skin and environmental contamination [8–10]. These factors ultimately result in greater transmission to HCWs and to other patients. In addition, exposure to antimicrobials reduces natural microflora, giving MDRO an advantage for colonization. An agent-based simulation model of an out-patient dialysis unit was therefore developed to quantify the contribution of SS1 and SS2 to the spread of MDRO, as well as the contribution of HCW and environmental contamination, and exposure to the hospital setting where MDRO acquisition frequently occurs [3].

The main findings of this study were that increasing the size of the SS2 population, via increasing antimicrobial exposure, resulted in a substantial rise in the prevalence of MDRO. For example, increasing the number of antimicrobial courses from once per year to four times per year increased the MDRO prevalence from 10% to 38%, and increasing the duration of antimicrobial exposure during a course of treatment by 7 days increased the overall MDRO prevalence by 5%.

These findings emphasize the need to limit antimicrobial exposure in dialysis units, a major risk factor for the emergence and spread of MDRO. Studies have shown that over 30% of antimicrobials administered in CHD units are not indicated [13]. Hospital-based antimicrobial stewardship programs are very effective in decreasing inappropriate antimicrobial prescribing. Developing such a program, which specifically targets the dialysis unit, would have beneficial effects in curtailing the ongoing rise of MDRO in the CHD population [17]. The substantial contribution of the SS2 subgroup to MDRO dissemination when antimicrobial use increases also suggests that infection control precautions should be implemented in this subgroup in order to limit spread and *de novo* acquisition by other patients.

Although environmental contamination of dialysis chairs and machines has been documented, its role in MDRO transmission has not been clearly quantified [2,18]. Our model suggests that reducing environmental contamination in the dialysis unit markedly reduces the average percentages of MDRO-colonized patients. For example, improving decontamination by 30% reduced the prevalence of MDRO by 10%. This corroborates a result from earlier work by Hotchkiss *et al*. who found that environmental decontamination markedly reduces MDRO transmission in the dialysis unit [19]. Efforts to improve compliance with decontamination guidelines should therefore be emphasized in dialysis units [7]. We note that antimicrobial exposure causes selective pressure on endogenous flora that allows for *de novo* acquisition due to contact with a contaminated environment. Again, reduction of antimicrobial exposure would ameliorate this problem.

Decreasing the influx of MDRO into the dialysis units by reducing in-hospital MDRO acquisition and by improving compliance with hand hygiene also decreased transmission. However, even with 100% compliance with the latter, 13.4% of patients remained colonized with MDRO. Although the hands of HCW are among the main vectors of MDRO spread, transmission of MDRO occurs through numerous paths, including a contaminated environment and hospital-acquired colonization.

This model quantifies the impact of various factors contributing to the spread of MDRO in dialysis units. MDRO transmission is multifactorial and therefore targeting only one factor is not sufficient. Current recommendations emphasize the importance of the SS1 subgroup, hand hygiene, and environmental decontamination. The findings of this model suggest that additional efforts should target the SS2 subgroup of patients, by limiting antimicrobial exposure and placing this subgroup of CHD patients on additional contact precautions, as are currently implemented on the SS1 group.
